# Equity in maternal health outcomes in a middle-income urban setting: a cohort study

**DOI:** 10.1186/s12978-019-0736-3

**Published:** 2019-06-18

**Authors:** Amanda De Groot, Lisanne Van de Munt, Daniel Boateng, Ary I. Savitri, Edward Antwi, Nienke Bolten, Kerstin Klipstein-Grobusch, Cuno S. P. M. Uiterwaal, Joyce L. Browne

**Affiliations:** 1 Midwifery Academy, Amsterdam, the Netherlands; 2 Julius Global Health, Julius Center for Health Sciences and Primary Care, University Medical Center Utrecht, Utrecht University, Utrecht, The Netherlands; 3 School of Public Health, Kwame Nkrumah University of Science and Technology, Kumasi, Ghana; 4 Ghana Health Service, Accra, Ghana; 5 Division of Epidemiology & Biostatistics, School of Public Health, Faculty of Health Sciences, University of the Witwatersrand, Johannesburg, South Africa

**Keywords:** Equity, Maternal health, Low- and middle income countries, Socio-economic status

## Abstract

**Background:**

Low socioeconomic status (SES) is associated with more adverse perinatal health outcomes, risk factors and lower access to and use of maternal health care services. However, evidence for the association between SES and maternal health outcomes is limited, particularly for middle-income countries like sub-Saharan Ghana. We assessed the association between parental SES and adverse maternal and perinatal outcomes of Ghanaian women during pregnancy, delivery and the postpartum period.

**Methods:**

A prospective cohort study of 1010 women of two public hospitals in Accra, Ghana (2012–2014). SES was proxied by maternal and paternal education, wealth and employment status. The association of SES with maternal and perinatal outcomes was analyzed with multivariable logistic and linear regression.

**Results:**

The analysis included 790 women with information on pregnancy outcomes. Average age was 28.2 years (standard deviation, SD 5.0). Over a third (*n* = 292, 37.0%) had low SES, 176 (22.3%) were classified to have high SES using the assets index. Nearly half (*n* = 374, 47.3%) of women had lower secondary school or vocational training as highest education level. Compared to women with middle assets SES, women with low assets SES were at higher risk for miscarriage (odds ratio, OR 1.61, 95% CI 1.06 to 2.45) and instrumental delivery (OR 1.74, 95% CI 1.03 to 2.94), but this association was not observed for the other SES proxies. For any of the maternal or perinatal outcomes and SES proxies, no other statistically significant differences were found.

**Conclusion:**

Women attending public maternal health care services in urban Ghana had overall equitable maternal and perinatal health outcomes, with the exception of a higher risk of miscarriage and instrumental delivery associated with low assets SES. This suggests known associations between SES, risk factors and outcomes could be mitigated with universal and accessible maternal health services.

**Electronic supplementary material:**

The online version of this article (10.1186/s12978-019-0736-3) contains supplementary material, which is available to authorized users.

## Plain English summary

Low socioeconomic status (SES) is associated with lower access to and use of maternal health care services, risk factors and poorer pregnancy outcomes for mother and child.

What did we study? We assessed if there was an association between SES (maternal education, paternal education, wealth and maternal employment status) and adverse maternal and perinatal outcomes of Ghanaian women during pregnancy, delivery and the postpartum period. We studied this in a group of 1010 pregnant women in two public hospitals in Accra, Ghana (2012–2014).

What did we find out? The analysis included 790 women with information on pregnancy outcomes. Over a third (*n* = 292, 37.0%) had low SES and 176 (22.3%) were classified to have high SES using the assets index. Nearly half (*n* = 374, 47.3%) of women had lower secondary school or vocational training as their highest educational level. Women with low assets SES had a higher risk for miscarriage and instrumental delivery (by vacuum, forceps, or Cesarean section) compared to women with middle assets SES. No other differences were found for any of the maternal or perinatal outcomes.

What do we conclude from these findings? Women who attend public maternal health care services in urban Ghana had comparable - equitable - maternal and perinatal health outcomes. This suggests that the known and previously described relationship between low SES, risk factors and poorer outcomes could be mitigated with universal and accessible maternal health services – as is the case in this setting.

## Background

Substantial inequities in maternal and perinatal health exists between and within countries, caused by societal contexts including health care systems and environmental circumstances [1, 2]. Within the Sustainable Development Goals (SDGs), improving maternal health (SDG 3.1), perinatal health (SDG 3.2) and reducing inequalities (SDG 10) are important priorities [3]. Yet, in many low- and middle-income countries (LMICs), substantial efforts will be required to achieve the SDGs’ ambitions by 2030.

Socio-economic status (SES) is the relative societal position of a person within a particular population [2]. Commonly used indicators of SES are level of education, employment, income and wealth [4]. Low SES is associated with increased exposure to health risks, lower access to and use of health care and poorer health outcomes [2]. Globally, maternal mortality ratios differ substantially between and within countries due to differences in SES [1]. Between global regions, maternal mortality ratios range between 12 (range 11–14) per 100.000 live births for high-resource settings and 546 (range 511–652) in sub-Saharan Africa in 2015 [5]. Similarly, within countries women with a high SES often have better maternal and perinatal health outcomes [1].

The mechanisms in which socio-economic status affects health are related to health seeking behavior, differences in quality of care received and a priori risk differences [6]. Higher educated women are more likely to know better when and how to seek appropriate health care compared to women with lower education [7]. Women who are poor and have lower or no education have lower antenatal, facility-based delivery and postnatal attendance rates, and receive less often care from skilled health workers, such as midwifes [6, 7] A priori risk differences by SES could be due to varied exposures to smoking, substance abuse, nutritional status, occupational health hazards or domestic violence across SES groups [6, 7].

Women with a lower SES are at increased risk of adverse perinatal outcomes including preterm birth, low birth weight, intra-uterine growth restriction, asphyxia and neonatal mortality [1, 8–13]. As preterm birth is the main cause of perinatal mortality and morbidity [8, 11], and premature babies are at increased risk of behavioral problems, respiratory and gastrointestinal complications and neurodevelopmental impairments including cerebral palsy, mental retardation and sensory deficits [11], SES affects health outcomes across generations.

While associations between low SES, maternal health services use as well as perinatal health outcomes are established [1], the evidence on the impact of low SES on maternal outcomes is relatively limited, especially in low- and middle income country setting [11, 12, 14]. Therefore, this study aimed to explore the influence of SES on maternal and perinatal outcomes in an urban region in a middle-income country, Ghana.

## Methods

### Study design and setting

This prospective cohort study was developed to assess factors related to maternal and perinatal outcomes of pregnant Ghanaian women, as described in detail elsewhere [15, 16]. Ghana has maternal mortality of 219 per 100.000 live births in 2015 and is classified as a middle-income country with an above median Human Development Index [17, 18]. The study was conducted at two outpatient departments (OPDs) of public hospitals in Accra, Ghana: the Maamobi General Hospital and Ridge Regional Hospital.

The Accra Metropolis is one of the local government districts of the Greater Accra Region of Ghana. The Greater Accra Region is the most densely populated region in Ghana and 90% urban, compared to 50% national urban residence [19]. The population growth in Accra is the highest in the country – primarily through migration for relatively better employment opportunities and the region has the lowest number of children born per woman. The Greater Accra Region has the lowest poverty levels in the country. Pregnant women in Ghana receive universal health insurance through the national health insurance scheme (NHIS) [20].

### Participants

Data from 1101 adult women were collected in the Accra Metropolis in Ghana from July 2012 to March 2014. Women were eligible for participation if they were over 18 years old, less than 17 weeks pregnant. Women with known pre-existent hypertension were excluded, because the initial aim of the cohort was to assess the incidence of gestational hypertension.

### Main exposure variable of interest

Inequities in health outcomes were assessed based on participants socio economic status (SES). Four proxies were used to estimate SES: maternal and paternal education, wealth index and employment status. Level of maternal and paternal education was classified into: (1) no education or primary school, (2) lower secondary school or vocational training, and (3) senior secondary school, professional school or higher tertiary education. This classification was both conceptually and data driven (i.e. sufficiently large categories of women whose education level was considered comparable). An asset (or wealth) index (range of − 10 to 20) was obtained through a principle component analysis (PCA) of various household assets and household characteristics. As such, the index estimates the relative wealth of a household by looking at their living conditions and items the household owns, allowing for differentiation of SES status within this population as described by Vyas and Kumaranayake [21]. The variables included in the PCA were presence and quantity of: irons, refrigerators, televisions, VCDDVD set, radio, landline phone, mobile phone, computer, generator, fan, mattresses or beds, watch/clock, sewing machine, modern stove, bicycle, motorcycle, car or truck and bednets. Household characteristics were also included in the PCA: whether any of the household members owned the house, the number of rooms in the house, materials of floors and roof, kind of toilet facilities, fuel used for cooking and where the household accessed water. The index was both used as a continuous variable and categorized according to quintiles: (1) low (lowest two quintiles), (2) middle (third and fourth quintiles), and (3) high (highest quintile), as described elsewhere [15, 16]. Employment was classified into (1) informal sector employment and (2) formal sector employment.

*Other exposure variables: demographics and anthropometry*Other covariates included woman’s age in years; body mass index (BMI) (m/kg^2^) based on measured weight and height; parity (0–1, 2–3, ≥4); gestational age based on ultrasound: first trimester (< 13 weeks), second trimester (≥13 weeks); area of birth (Ghana urban, Ghana rural, West African country); area of residence (Accra metropolitan area, other urban area, peri-urban and rural area); ethnicity (Akan, Hausa, Ewe, Ga Ga-Dangme, other); religion (Christian, Islam) and marital status (single or widowed, married, engaged or living together).

### Outcomes

#### Maternal outcomes measurements and classifications

Gestational hypertension (GH) was defined according to the ISSHP definition as “a systolic blood pressure ≥140 mmHg and/or a diastolic blood pressure ≥90 mmHg after 20 weeks gestation, measured twice, with women who previously had normal blood pressure” [22]. Blood pressure was measured according to Korotkov V according to hospital protocols [15, 16]. Pre-eclampsia (PE) was defined as “the combination of pregnancy induced hypertension with proteinuria (≥300 mg/ 24 hours), or minimal 1+ on a dipstick” [22]. Because of the low numbers of women GH and PE in the cohort, these two outcomes were combined and further referred to as hypertensive disorders (yes/no) of pregnancy.

Postpartum hemorrhage (PPH) was defined as ‘blood loss more than 500 ml in the first 24 h after delivery [23]. The total blood loss was visually estimated by the midwives of the two hospitals. PPH was categorized into two groups based on the estimated amount of blood loss; < 500 ml and ≥ 500 ml.

Maternal mortality was defined as “direct mortality due to complications of pregnancy, delivery, and puerperium”.

Mode of delivery was defined as either spontaneous vaginal delivery or instrumental delivery including cesarean section (CS) and assisted delivery (vacuum or forceps). Because of the low numbers of cesarean and assisted delivery, these categories were combined to allow for higher numbers of women per category.

#### Perinatal outcomes

WHO definitions were used for miscarriage, perinatal mortality, stillbirth, and preterm birth, as previously described [15, 16]. Apgar score was evaluated at 5 min after birth based on heart rate, respiratory effort, muscle tone, reflex irritability, and skin color. A score of ≥7 was considered normal. Birth weight was analyzed both as continuous and categorical variables (low birth weight (< 2500 g), normal birth weight (≥2500 and ≤ 4000 g), or macrosomia (> 4000 g)).

#### Data sources

Women were recruited at the their first antenatal care (ANC) visit, where baseline independent variable data was collected by seven trained research assistants. The assistants used a structured questionnaire for socio-demographic characteristics (area of birth, area of residence, ethnical groups, religion and marital status), socio-economic characteristics (level of education, economic activity, assets, and household characteristics), and health status including obstetric history. Pregnancy outcomes, both maternal and neonatal, were obtained from the patient registers available at the two participating hospitals. The information contained in the antenatal record books (which women keep themselves throughout their pregnancy) was also used for prenatal information. Data collection occurred at enrolment, after delivery and at 6 weeks postpartum during the postnatal visit. Prior to the start of the study, questionnaires were validated.

Data was entered by trained data clerks using EpiDataEntry software (EpiData Association, Odense, Denmark, 2010). The data was validated by double entry and checked for missing data.

### Data analysis

Participant characteristics were analyzed descriptively with frequencies (%) and means (standard deviation, SD) where appropriate, by categories of SES. Group (SES) differences were assessed by chi-square test (or Fisher’s exact) and one-way ANOVA for categorical and continuous variables respectively.

Depending on the type of outcome variables (binary or continuous), logistic or linear regression analyses were used. Odds ratios (OR) and linear coefficients with corresponding 95% confidence intervals (CI) and two-sided *p*-values were respectively reported. In adjusted models, regression analysis were controlled for maternal age and body mass index (BMI). For SES estimates with multiple levels, the middle SES group was used as reference. For all analyses, participating women had to have at least one recorded maternal or perinatal outcome. If not, women were considered loss to follow up and not included in the analysis. Missing data was considered missing completely at random (MCAR) and complete-case analysis performed. All analyses were performed using IBM SPSS Statistics version 22 [24].

### Ethics

This study was approved by the Ghana Health Services Ethical Review Committee (GHS-ERC 07/9/11). All participants provided (written or thumb-printed) informed consent.

## Results

**Fig. 1:**
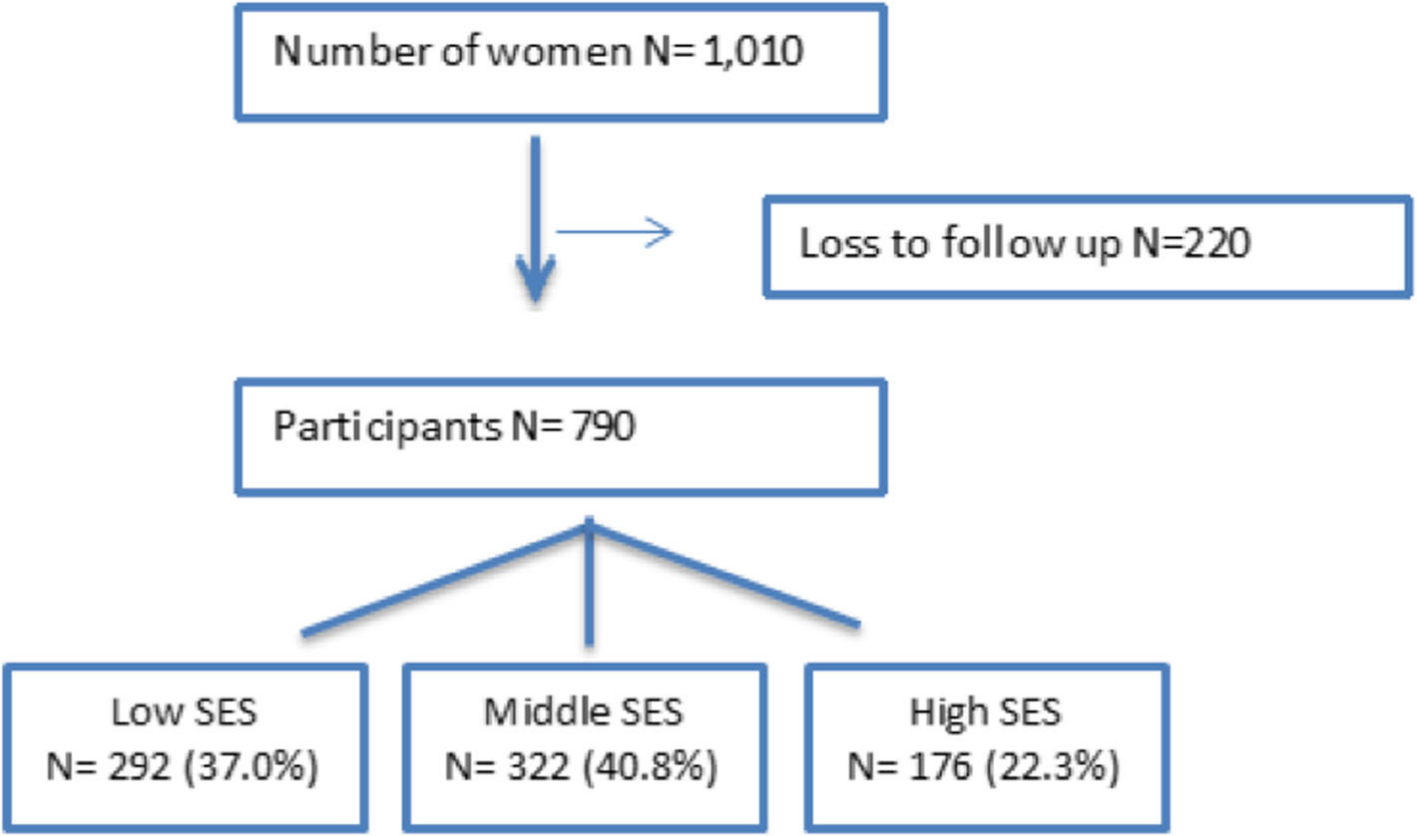
Flowchart of inclusion of participants in this cohort study

Of all enrolled 1010 women, SES information was available. For 790 women there was at least one outcome measurement available (Fig. 1), thus 21.8% were considered lost to follow-up. No significant differences were seen in the general characteristics of women who were included in the study as compared to those who were excluded or lost to follow up.

Table 1 presents pregnancy, demographic and socio- economic characteristics of participants. Over a third (*n* = 292, 37.0%) of women had low SES, 176 (22.3%) were classified to have high SES using the assets index. Nearly half (*n* = 374, 47.3%) of women had lower secondary school or vocational training as highest education level. The majority of women in the study (69.9%) were pregnant for either the first or second time. Women with a low SES were on average younger, more often in their second trimester at antenatal booking, more often originally from rural areas, currently residing in the Accra metropolis, less likely from Akan and Ga ethnic groups, and less likely to be married.

**Table 1:** Pregnancy, demographic and socio-economic characteristics of women in Accra, Ghana

		Cohort including women lost to follow up						Cohort excluding women lost to follow up					
				Socioeconomic status by assets						Socioeconomic status by assets			
		N	All	Low *N* = 406	Middle *N* = 402	High *N* = 202	*P* value	N	All	Low N = 292	Middle *N* = 322	High *N* = 176	*P* value
Mean age (yr, SD)		1.010	28.0 (5.1)	27.3 (5.4)	28.2 (4.8)	29.0 (5.0)	0.00	790	28.2 (5.0)	27.5 (5.2)	28.3 (4.7)	28.9 (5.1)	0.01
Mean BMI at ANC booking (kg m^−2^, SD)		1.000	25.4 (4.7)	25.0 (4.5)	25.6 (4.8)	25.8 (4.7)	0.10	785	25.5 (4.7)	25.1 (4.5)	25.8 (4.8)	25.8 (4.8)	0.18
Mean gestational age at delivery (wk, SD)		739	39.1 (1.9)	39.1 (1.9)	39.1 (1.8)	39.1 (2.1)	0.91	739	39.1 (1.9)	39.1 (1.9)	39.1 (1.8)	39.1 (2.1)	0.91
Parity (n,%)	0–1	1.010	724 (72)	291 (72)	296 (74)	137 (68)	0.13	790	552 (70)	200 (69)	233 (72)	119 (68)	0.43
	2–3		263 (26)	106 (26)	101 (25)	56 (28)			219 (28)	85 (29)	84 (26)	50 (28)	
	≥4		23 (2)	9 (2)	5 (1)	9 (5)			19 (2)	7 (2)	5 (2)	7 (4)	
Gestational age 1st ANC (n,%)	1st trimester (<13w)	1.001	556 (56)	203 (51)	231 (58)	122 (61)	0.02	785	423 (54)	140 (48)	182 (57)	101 (58)	0.05
	2nd trimester (≥13w)		445 (45)	199 (50)	168 (42)	78 (39)			362 (46)	150 (52)	139 (43)	73 (42)	
Area of birth (n,%)	Ghana urban	1.010	795 (79)	273 (67)	338 (84)	184 (91)	0.00	790	634 (80)	201 (69)	271 (84)	162 (92)	0.00
	Ghana rural		196 (19)	122 (30)	60 (15)	14 (7)			142 (18)	85 (29)	47 (15)	10 (6)	
	West African country		19 (2)	11 (3	4 (1)	4 (2)			14 (2)	6 (2)	4 (1)	4 (2)	
Area of residence (n,%)	Accra metropolitan	1.010	788 (78)	322 (79)	307 (76)	159 (79)	0.15	790	619 (78)	235 (81)	247 (77)	137 (78)	0.29
	Other urban		189 (19)	68 (17)	87 (22)	34 (17)			145 (18)	46 (16)	68 (21)	31 (18)	
	Peri- urban and rural		33 (3)	16 (4)	8 (2)	9 (5)			26 (3)	11 (4)	7 (2)	8 (5)	
Ethnical groups (n,%)	Akan	1.010	359 (36)	128 (32)	148 (37)	83 (41)	0.00	790	285 (36)	96 (33)	119 (37)	70 (40)	0.01
	Hausa		197 (20)	80 (20)	85 (21)	32 (16)			150 (19)	57 (20)	67 (21)	26 (15)	
	Ewe		213 (21)	91 (22)	84 (21)	38 (19)			161 (20)	60 (21)	67 (21)	34 (19)	
	Ga, Ga- Dangme		97 (10)	31 (8)	33 (8)	33 (16)			81 (10)	25 (9)	26 (8)	30 (17)	
	Other		144 (14)	76 (19)	52 (13)	16 (8)			113 (14)	54 (19)	43 (13)	16 (9)	
Religion (n,%)	Christianity	1.010	724 (72)	283 (70)	285 (71)	156 (77)	0.14	790	571 (72)	203 (70)	230 (71)	138 (78)	0.10
	Islam		286 (28)	123 (30)	117 (29)	46 (23)			219 (28)	89 (31)	92 (29)	38 (22)	
Marital status (n,%)	Single, widowed	1.010	190 (19)	95 (23)	59 (15)	36 (18)	0.00	790	141 (18)	69 (24)	40 (12)	32 (18)	0.00
	Married		605 (60)	210 (52)	257 (64)	138 (68)			492 (62)	157 (54)	217 (67)	118 (67)	
	Engaged, living together		215 (21)	101 (25)	86 (21)	28 (14)			157 (20)	66 (23)	65 (20)	26 (15)	

### Perinatal and maternal outcomes

Perinatal and maternal outcomes are shown by SES categories in Table 2. Among the participants, one maternal death (0.1%) occurred with an unknown cause. Out of 790 women analysed, 88% delivered spontaneous vaginally (*n* = 654), without complication and at term (preterm birth: 8%). About 17% (*n* = 136) experienced miscarriage and 1% (*n* = 11) perinatal mortality. The birth weight was on average 3122 g (SD 495.43), with 7% (*n* = 54/782) categorized as low birth weight and 5% (*n* = 42) as macrosomic. Nearly all infants had a good Apgar score (95%, 572/597). No associations between SES and maternal or perinatal outcomes were observed, except for a trend for miscarriage with lower SES (*p* = 0.07).

**Table 2:** Overview of perinatal and maternal outcomes of pregnant women in Accra, Ghana

				Socioeconomic status by assets			
		N	All (%)	Low N = 292	Middle N = 322	High N = 176	*P* value
Maternal outcomes							
Maternal mortality (n,%)	No	789	788 (99)	291 (100)	321 (99)	176 (100)	1.00
	Yes		1 (1)	0 (0)	1 (1)	0 (0)	
Hypertensive disorder of pregnancy (n,%)	No	789	716 (91)	268 (92)	292 (91)	156 (89)	0.52
	Yes		73 (9)	24 (8)	29 (9)	20 (11)	
PPH > 500 ml (n,%)	No	705	684 (97)	252 (97)	283 (98)	149 (96)	0.49
	Yes		21 (3)	9 (3)	6 (2)	6 (4)	
Delivery (n,%)	Spontaneous	790	698 (88)	254 (87)	293 (91)	151 (86)	0.15
	Instrumental or Cesarean		92 (12)	38 (13)	29 (9)	25 (14)	
Perinatal outcomes							
Miscarriage (n,%)	No	790	654 (83)	230 (79)	274 (85)	150 (85)	0.07
	Yes		136 (17)	62 (21)	48 (15)	26 (15)	
Perinatal mortality (n,%)	No	790	779 (99)	288 (99)	317 (98)	174 (99)	1.00
	Yes		11 (1)	4 (1)	5 (2)	2 (1)	
Stillbirth (n,%)	No	790	784 (99)	289 (99)	321 (99)	174 (99)	0.45
	Yes		6 (1)	3 (1)	1 (1)	2 (1)	
Birth weight (g) (n,%)	< 2500	782	54 (7)	22 (8)	18 (6)	14 (8)	0.31
	2500–4000		681 (88)	253 (89)	276 (87)	152 (87)	
	> 4000		42 (5)	11 (4)	23 (7)	8 (6)	
Preterm birth (n,%)	No	739	682 (92)	248 (91)	283 (93)	151 (94)	0.49
	Yes		57 (8)	25 (9)	22 (7)	10 (6)	
Apgar score after 5 min (n,%)	< 7	597	25 (4)	13 (6)	7 (3)	5 (4)	0.29
	≥7		572 (96)	210 (94)	231 (97)	131 (96)	
Birth weight (g) (mean, SD)		777	3123 (495)	3092 (479)	3151 (490)	3122 (531)	0.34

The associations between SES (as measured by household assets/wealth) and maternal and perinatal outcomes are shown in Table 3. Compared to women with middle SES, women with a low SES had an increased risk to have a miscarriage (adjusted odds ratio, aOR 1.61, 95% CI 1.06 to 2.45). For women with a low SES, instrumental delivery occurred more often compared to women with middle SES (aOR 1.74, 95% CI 1.03 to 2.94). For other maternal and perinatal outcomes no significant differences occurred.

**Table 3:** Association of SES category by assets and maternal and perinatal outcomes for pregnant women in Accra, Ghana

	Model	Socioeconomic status by assets						
		Low		Middle	High		SES score	
		OR (95% CI)	*P* value	*Ref.*	OR (95% CI)	*P* value	OR (95% CI)	*P* value
Maternal outcomes								
Hypertensive disorder of pregnancy	Crude	0.90 (0.51–1.59)	0.72	1.00	1.29 (0.71–2.36)	0.41	1.08 (0.99–1.18)	0.10
	Adjusted	0.99 (0.55–1.76)	0.97	*1.00*	1.10 (0.58–2.09)	0.78	1.04 (0.94–1.15)	0.46
PPH > 500 ml	Crude	1.69 (0.59–4.78)	0.33	1.00	1.90 (0.60–5.99)	0.27	1.04 (0.88–1.22)	0.69
	Adjusted	1.85 (0.65–5.31)	0.25		1.87 (0.59–5.92)	0.29	1.02 (0.85–1.22)	0.84
Vaginal vs instrumental/CS delivery	Crude	1.51 (0.91–2.52)	0.11		1.67 (0.95–2.96)	0.08	1.00 (0.92–1.10)	0.96
	Adjusted	1.74 (1.03–2.94)	0.04*		1.65 (0.92–2.98)	0.09	0.98 (0.89–1.08)	0.63
Perinatal outcomes								
Miscarriage	Crude	1.54 (1.02–2.33)	0.04*		0.97 (0.59–1.66)	0.97	0.93 (0.85–1.01)	0.07
	Adjusted	1.61 (1.06–2.45)	0.03*		1.02 (0.60–1.71)	0.95	0.92 (0.85–1.00)	0.06
Perinatal mortality	Crude	0.88 (0.23–3.31)	0.85		0.73 (0.14–3.80)	0.71	0.93 (0.71–1.22)	0.59
	Adjusted	0.84 (0.22–3.16)	0.79		0.74 (0.14–3.87)	0.72	0.94 (0.72–1.23)	0.66
Stillbirth	Crude	3.33 (0.35–32.21)	0.30		3.69 (0.33–40.98)	0.29	0.94 (0.65–1.35)	0.72
	Adjusted	3.25 (0.33–31.56)	0.31		3.48 (0.31–38.96)	0.31	0.94 (0.65–1.34)	0.73
Low birthweight	Crude	1.38 (0.72–2.63)	0.33		1.46 (0.71–3.01)	0.31	1.02 (0.91–1.14)	0.77
	Adjusted	1.33 (0.70–2.55)	0.38		1.50 (0.73–3.11)	0.27	1.03 (0.92–1.15)	0.63
Macrosomia	Crude	0.51 (0.25–1.07)	0.07		0.62 (0.27–1.41)	0.25	1.01 (0.89–1.15)	0.83
	Adjusted	0.55 (0.26–1.14)	0.11		0.60 (0.26–1.38)	0.23	1.00 (0.88–1.14)	0.99
Preterm birth	Crude	1.30 (0.71–2.36)	0.39		0.85 (0.39–1.85)	0.68	0.96 (0.85–1.08)	0.52
	Adjusted	1.35 (0.74–2.46)	0.33		0.85 (0.39–1.84)	0.67	0.95 (0.85–1.08)	0.45
Apgar score < 7 after 5 min	Crude	2.04 (0.80–5.22)	0.14		1.26 (0.39–4.05)	0.70	0.95 (0.79–1.13)	0.54
	Adjusted	2.00 (0.78–5.13)	0.15		1.28 (0.40–4.12)	0.68	0.95 (0.79–1.14)	0.58
		B (CI 95%)	*P* value		B (CI 95%)	*P* value	B (CI 95%)	*P* value
Birthweight (g)	Crude	−59.50 (− 138.80–19.81)	0.14		−28.69 (− 120.44–63.06)	0.54	4.13 (−10.30–18.55)	0.58
	Adjusted	−46.15 (− 125.24–32.95)	0.25		−28.66 (−120.32–63.00)		1.24 (−13.31–15.79)	0.87

*OR* odds ratio, *PPH* post partum hemorrhage, *CS* cesarean section. *p < 0.05

For the other proxies of SES (Additional file 1: Tables S1, S2 and S3) for maternal education, paternal education and employment, associations were inconsistent. No association between maternal education or employment status, but a higher level of paternal education was associated with a trend towards an increased risk of hypertensive disorders of pregnancy (aOR 1.71, 95%CI 0.98–2.98).

## Discussion

This study shows an increased risk for miscarriage and instrumental or CS delivery for women with a low SES estimated by wealth status compared to middle SES. No further associations between other socio-economic status proxies and maternal and perinatal outcomes in this urban population of Ghanaian women attending the public health facilities for antenatal health in the first half of their pregnancy were observed.

Previous studies have observed differences in particularly SES and use of antenatal care, facility based delivery and postnatal care, as well as associations between SES and perinatal outcomes [1]. How these translate in actual health outcomes have been less intensively studied. An explanation for the lack of consistent direction of this study (i.e. adverse outcomes associated with SES) could be the context-specific characteristics in Ghana, which may promote equity in outcomes. Ghana is a middle-income country and this study was conducted in an urban area with quality health services available, reflected in high overall ANC attendance and facility-based delivery rates. However, when disaggregated into SES levels – skilled delivery and ANC attendance are lower for low and middle SES women compared to high SES women: 35% vs 100% and 75 vs 100%, respectively. All women in Ghana receive insurance during their pregnancy, which has previously been shown to be associated with a higher maternal health services use across SES status [20]. As such, it is possible that the Ghanaian public health services mitigate against the translation between inequities in *socio-economic status* to inequities in maternal and perinatal *outcomes*.

Other studies conducted about the association between SES and maternal and perinatal health outcomes in Ghana showed varying observations: a previous WHO study showed few differences between no education and secondary or higher education on infant mortality [2]. Another cross-sectional study performed in (peri-)urban Cape Coast, Ghana observed pronounced differences of SES (defined as area of residence, education and income) on birth weight in a cohort of 559 women. This was associated with a 292 g (95% CI, − 440 to − 145) reduction in birth weight at a mean birth weight of 3026.7 g [11]. Similarly, another sub-Saharan African country retrospective study among 11,872 participants from predominantly rural Ethiopia observed that having a higher education level and high household income was a protective factor against getting a low birth weight baby with respectively ORs between 0.42 (95% CI, 0.29 to 0.59) and 0.22 (95% CI, 0.00 to 0.50) [25]. The differences in area of residence and/or health care system between the study populations in these studies may explain the different results, and could be explored in future research.

Our finding that the risk of miscarriage in this study was significantly related to SES by assets - but not by other proxies, we cannot readily explain. One possible explanation could be that women with low SES in urban settings are more exposed to psychosocial stress as a result of poverty. An association between psychosocial stress and miscarriage was recently described [26, 27]. In addition, most low SES women were migrants from rural areas, are of other ethnic groups (slightly more Akan and Ga/Ga-Gangme in high SES group), with low educational level and are therefore more likely to be engaged in labour intensive occupations, which may have exposed them to a risk to miscarry. The overall rate of miscarriage in our study (17%) is higher than previously reported in the Ghana Maternal Health Survey of 2017 (12%) [28], possibly a reflection of the early gestational age enrolment of this prospective cohort (< 17 weeks of gestation), compared to the average gestational age at first antenatal visit (> 20 weeks).

We choose to approach equities in health outcomes by assessing how various maternal health outcomes differ between various socio-economic groups. Although beyond the scope of these analyses, it should be considered that other determinants, such as based on demohraphy and geography, can also result in inequities in health. SES was approached with various proxies as these may measure different and complimentary aspects. Assets are considered a more long-term indicator of wealth, and less volatile than income, especially in populations with a high prevalence of informal sector labor [29, 30]. Although education is similarly a ‘longer acting’ proxy, higher levels of education do not always translate to higher household wealth with high levels of (youth) unemployment. One other possible reason why specifically assets were associated with increased risk of miscarriage could be related to the lifestyle and household factors such as the use of biomass fuel or charcoal as cooking fuels [11].

### Strengths and limitations

A strength of this study is the large size of the cohort with 1010 women enrolled in early pregnancy and a high study completion rate of about 80% - reflecting extensive efforts of this study to follow women up. As previously reported, women lost to follow up were more likely to be nulliparous and with a lower SES, and if these would have had a higher risk of adverse events – the results could have been attenuated in this study. Similarly, women who experienced a miscarriage may also more likely to report back, resulting in a higher attrition of women with this adverse outcome in the overall study. The data was prospectively collected, which reduces the risk of selection bias, and there was optimal control of data measurement and registration because of the questionnaires that was linked to health services provision. Women were included in early pregnancy, allowing for an assessment of their health status in early pregnancy. And whilst status of SES can change over the course of pregnancy (i.e. wealth), the combination of various SES proxies and consistency of results, suggest validity of the observations. A particular strength is that this study is one of few studies investigating the association between SES and maternal and perinatal outcomes in a middle-income country with a low prevalence of (second-hand) smoking (< 1% of women) [31]. As smoking is among one of the strongest mechanisms between SES and health outcomes, our study allowed for an unconfounded assessment in this regard.

Some considerations must be made in the interpretation of the results. The population enrolled in this study attended antenatal care relatively early (< 17 weeks pregnancy) for Ghanaian standards. This could have affected the results in two directions: women more aware of recommendations about early first antenatal care (and possible general advice about healthy pregnancies) visits may have been overrepresented in our population. As some outcomes occurred rarely, for example maternal death, this could therefore not be analyzed. Another limitation may be that relatively few women with extreme exposure of low SES were included: this study only included women who attended ANC services, excluding those who did not enroll in ANC services due to lack of money, access to transport or who attended much later in pregnancy. However, with advent of NHIS and the context of Accra, even for women with low SES, barriers to access ANC services or transportation are relatively low [32]. Similarly, women with high SES may prefer go to a private hospital with better health care facilities. Although this limits the generalizability of the findings beyond an urban area in middle-income countries with relatively good public health facilities, the results do show how equity can be promoted within this system.

## Conclusion and recommendations

This study suggests that improved access to maternal healthcare as provided under the Ghana NHIS is associated with equity in maternal health outcomes overall in urban Ghana. A higher risk of miscarriage and instrumental delivery was observed with low SES based on assets. However, as the population of the study was limited to women attending public antenatal care in a highly urbanized area, those with a low SES and at the highest SES categories may have been precluded. This study was not large enough to assess the impact on rarer pregnancy outcomes such as severe morbidity and maternal or perinatal death and this could be an area of continued research. In addition, future research could explore the mechanisms to promote equity within in a health care system.

## Additional file

Additional file 1: **Table S1.** Association of SES category by maternal education and maternal and perinatal outcomes for pregnant women in Accra, Ghana. **Table S2.** Association of SES category by paternal education and maternal and perinatal outcomes for pregnant women in Accra, Ghana. **Table S3.** Association of SES category by employment and maternal and perinatal outcomes for pregnant women in Accra, Ghana (DOCX 31 kb)

## Abbreviations

ANC: Antenatal care
BMI: Body mass index
CI: Confidence interval
CS: Cesarean section
GH: Gestational Hypertension
NHIS: National health insurance scheme
OR: Odds ratios
PCA: Principle component analysis
PE: Preeclampsia
PPH: Postpartum haemorrhage
SD: Standard deviation
SDGs: Sustainable Development Goals
SES: socio-economic status
WHO: World Health Organization

## References

[1] Boerma T, Requejo J, Victora CG, Amouzou A, George A, Agyepong I (2018). Countdown to 2030: tracking progress towards universal coverage for reproductive, maternal, newborn, and child health. Lancet..

[2] Closing the gap in a generation: health equity through action on the social, determinants of health. Final report of the commission on social determinants of health. Closing the gap in a generation. Geneva; 2008. Available from: https://apps.who.int/iris/bitstream/handle/10665/43943/9789241563703_eng.pdf?sequence=1&isAllowed=y. Accessed 1 Aug 2018.

[3] UN General Assembly (70th session) (2015). Transforming our world: the 2030 agenda for sustainable development, resolution adopted by the general assembly. A/70/L.1.

[4] Bartley M (2004). Health inequality: an introduction to theories, concepts, and methods.

[5] Alkema L, Chou D, Hogan D, Zhang S, Moller A-B, Gemmill A (2016). Global, regional, and national levels and trends in maternal mortality between 1990 and 2015, with scenario-based projections to 2030: a systematic analysis by the UN maternal mortality estimation inter-agency group. Lancet..

[6] Cutler D, Lleras-Muney A, Vogl T. In: Glied S, Smith PC, editors. Socioeconomic status and health: dimensions and mechanisms: Oxford Handb. Heal. Econ. Oxford: Oxford Handbooks; 2011.

[7] UNFPA. Rich Mother, Poor Mother: the social determinants of maternal death and disability. New York; 2012. Available from: https://www.unfpa.org/sites/default/files/resource-pdf/EN-SRH%20fact%20sheet-Poormother.pdf. Accessed 1 Aug 2018.

[8] Clayborne ZM, Giesbrecht GF, Bell RC, Tomfohr-Madsen LM (2017). Relations between neighbourhood socioeconomic status and birth outcomes are mediated by maternal weight. Soc Sci Med.

[9] Luo Z-C, Wilkins R, Kramer MS (2006). Fetal and infant health study Group of the Canadian Perinatal Surveillance System. Effect of neighbourhood income and maternal education on birth outcomes: a population-based study. CMAJ..

[10] Pickett KE, Ahern JE, Selvin S, Abrams B (2002). Neighborhood socioeconomic status, maternal race and preterm delivery: a case-control study. Ann Epidemiol.

[11] Amegah AK, Damptey OK, Sarpong GA, Duah E, Vervoorn DJ, Jaakkola JJK (2013). Malaria infection, poor nutrition and indoor air pollution mediate socioeconomic differences in adverse pregnancy outcomes in Cape Coast, Ghana. Kazembe L, editor. PLoS One.

[12] Alosaimi AN, Luoto R, Al Serouri AW, Nwaru BI, Mouniri H (2016). Measures of Maternal Socioeconomic Status in Yemen and Association with Maternal and Child Health Outcomes. Matern Child Health J.

[13] Almeida NKO, Pedreira CE, Almeida RMVR (2016). Impact of maternal education level on risk of low Apgar score. Public Health.

[14] Davies HR, Visser J, Tomlinson M, Rotherham-Borus MJ, LeRoux I, Gissane C (2012). An investigation into the influence of socioeconomic variables on gestational body mass index in pregnant women living in a peri-urban settlement, South Africa. Matern Child Health J.

[15] Van Der Linden EL, Browne JL, Vissers KM, Antwi E, Agyepong IA, Grobbee DE (2016). Maternal body mass index and adverse pregnancy outcomes: a Ghanaian cohort study. Obesity..

[16] Browne JL, Vissers KM, Antwi E, Srofenyoh EK, Van der Linden EL, Agyepong IA (2015). Perinatal outcomes after hypertensive disorders in pregnancy in a poor urban setting. Trop Med Int Heal.

[17] The World Bank. Ghana | Data. 2018. Available from: https://data.worldbank.org/country/Ghana. [cited 2018 Apr 5]

[18] WHO, UNICEF, UNFPA, World Bank Group and UNPDMMEI-AG. Maternal mortality in 1990–2015: Ghana. 2016. Available from: http://www.who.int/gho/maternal_health/countries/gha.pdf. Accessed 1 Aug 2018.

[19] Ghana Statistical Service. 2010 Population and housing census. Ghana Stat Serv Accra. 2012; Available from: http://www.statsghana.gov.gh/gssmain/storage/img/marqueeupdater/Census2010_Summary_report_of_final_results.pdf. Accessed 1 Aug 2018.

[20] Browne JL, Kayode GA, Arhinful D, Fidder SAJ, Grobbee DE, Klipstein-Grobusch K (2016). Health insurance determines antenatal, delivery and postnatal care utilisation: evidence from the Ghana demographic and health surveillance data. BMJ Open.

[21] Vyas S, Kumaranayake L (2006). Constructing socio-economic status indices: how to use principal components analysis. Health Policy Plan.

[22] Tranquilli a L, Dekker G, Magee L, Roberts J, Sibai BM, Steyn W (2014). The classification, diagnosis and management of the hypertensive disorders of pregnancy: a revised statement from the ISSHP. Pregnancy Hypertens.

[23] World Health Organization (2011). WHO recommendations for prevention and treatment of pre-eclampsia and eclampsia.

[24] IBM Corp. Released 2013. IBM SPSS Statistics for Windows, Version 22.0. Armonk: IBM Corp; 2013.

[25] Alemu T, Umeta M (2016). Prevalence and Predictors of Small Size Babies in Ethiopia: In-depth analysis of the Ethiopian demographic and health survey, 2011, Ethiop. Ethiop J Health Sci.

[26] Santiago CD, Wadsworth ME, Stump J (2011). Socioeconomic status, neighborhood disadvantage, and poverty-related stress: prospective effects on psychological syndromes among diverse low-income families. J Econ Psychol.

[27] Qu F, Wu Y, Zhu Y-H, Barry J, Ding T, Baio G (2017). The association between psychological stress and miscarriage: a systematic review and meta-analysis. Sci Rep.

[28] Ghana Statistical Service, Ghana Health Services and The DHS Program ICF. Maternal Health Survery 2017: Key Indicators Report. Available from: http://www2.statsghana.gov.gh/docfiles/PR95.pdf. Accessed 1 Aug 2018.

[29] Filmer D, Scott K (2012). Assessing asset indices. Demography.

[30] Howe LD, Galobardes B, Matijasevich A, Gordon D, Johnston D, Onwujekwe O (2012). Measuring socio-economic position for epidemiological studies in low- and middle-income countries: a methods of measurement in epidemiology paper. Int J Epidemiol.

[31] Owusu-Dabo E, Lewis S, McNeill A, Gilmore A, Britton J (2009). Smoking uptake and prevalence in Ghana. Tob Control.

[32] Singh K, Osei-Akoto I, Otchere F, Sodzi-Tettey S, Barrington C, Huang C (2015). Ghana’s National Health insurance scheme and maternal and child health: a mixed methods study. BMC Health Serv Res.

