# Supplementary material for: Equity in maternal health outcomes in a middle-income urban setting: a cohort study
Source: Reprod Health. 2019 Jun 18;16:84. doi: 10.1186/s12978-019-0736-3 (PMC6580627; doi:10.1186/s12978-019-0736-3)
Supplement: Supplementary file 1 — Table S1. Association of SES category by maternal education and maternal and perinatal outcomes for pregnant women in Accra, Ghana. Table S2. Association of SES category by paternal education and maternal and perinatal outcomes for pregnant women in Accra, Ghana. Table S3. Association of SES category by employment and maternal and perinatal outcomes for pregnant women in Accra, Ghana (DOCX 31 kb) [file 12978_2019_736_MOESM1_ESM.docx]

Table S1. Association of SES category by maternal education and maternal and perinatal outcomes for pregnant women in Accra, Ghana

|  |  | **Socioeconomic status by maternal education** | | |  | |
| --- | --- | --- | --- | --- | --- | --- |
|  |  | **No education/ primary school** | | **Lower secondary school/ vocational training** | **Senior secondary school/ professional school/ higher tertiary education** | |
|  | **Model** | **OR (95% CI)** | ***P* value** | ***Ref.*** | **OR (95% CI)** | ***P* value** |
| **Maternal outcomes** |  |  |  |  |  |  |
| Hypertensive disorder of pregnancy | Crude | 0.83 (0.43 - 1.63) | 0.60 |  | 1.30 (0.76 - 2.22) | 0.34 |
|  | Adjusted | 0.86 (0.43 - 1.74) | 0.69 |  | 1.27 90.73 - 2.23) | 0.40 |
| PPH >500 ml | Crude | 0.38 (0.08 - 1.75) | 0.21 |  | 1.14 (0.45 - 2.88) | 0.78 |
|  | Adjusted | 0.42 (0.09 - 1.94) | 0.27 |  | 1.19 (0.47 - 3.01) | 0.72 |
| Vaginal vs instrumental/CS delivery | Crude | 0.38 (0.08 - 1.75) | 0.22 |  | 1.13 (0.45 - 2.85) | 0.80 |
|  | Adjusted | 0.81 (0.44 - 1.50) | 0.51 |  | 1.05 (0.63 - 1.74) | 0.86 |
|  |  |  |  |  |  |  |
| **Perinatal outcomes** |  |  |  |  |  |  |
| Miscarriage | Crude | 1.32 (0.83 - 2.09) | 0.24 |  | 1.07 (0.70 - 1.65) | 0.77 |
|  | Adjusted | 1.36 (0.85 - 2.16) | 0.20 |  | 1.11 (0.72 - 1.71) | 0.65 |
| Perinatal mortality | Crude | 1.30 (0.31 - 5.48) | 0.73 |  | 0.93 (0.22 - 3.91) | 0.92 |
|  | Adjusted | 1.24 (0.29 - 5.26) | 0.77 |  | 0.91 (0.22 - 3.87) | 0.90 |
| Stillbirth | Crude | 4.34 (0.39 - 48.16) | 0.23 |  | 4.68 (0.48 - 45.27) | 0.18 |
|  | Adjusted | 4.23 (0.38 - 47.17) | 0.24 |  | 4.75 (0.49 - 46.12) | 0.18 |
| Low birthweight | Crude | 0.91 (0.42 - 1.96) | 0.81 |  | 1.41 (0.76 - 2.60) | 0.28 |
|  | Adjusted | 0.89 (0.41 - 1.92) | 0.77 |  | 1.41 (0.76 - 2.61) | 0.28 |
| Macrosomia | Crude | 0.85 (0.42 - 1.70) | 0.65 |  | 0.65 (0.33 - 1.28) | 0.21 |
|  | Adjusted | 0.90 (0.45 - 1.81) | 0.76 |  | 0.67 (0.34 - 1.32) | 0.25 |
| Preterm birth | Crude | 0.96 (0.49 - 1.91) | 0.91 |  | 0.89 (0.47 - 1.69) | 0.72 |
|  | Adjusted | 0.98 (0.49 - 1.94) | 0.95 |  | 0.91 (0.48 - 1.73) | 0.77 |
| Apgar score < 7  after 5 minutes | Crude | 1.99 (0.79 - 4.98) | 0.14 |  | 0.93 (0.33 - 2.58) | 0.88 |
|  | Adjusted | 1.95 (0.78 - 4.90) | 0.16 |  | 0.93 (0.33 - 2.58) | 0.88 |
|  |  |  |  |  |  |  |
|  |  | **B (CI 95%)** | ***P* value** |  | **B (CI 95%)** | ***P* value** |
| **Birthweight (g)** | Crude | -18.14 (-108.09 - 71.82) | 0.69 |  | -13.01 (-93.92 - 67.89) | 0.75 |
|  | Adjusted | -4.36 (-93.94 - 85.21) | 0.92 |  | -5.80 (-86.40 - 74.80) | 0.89 |

OR, odds ratio; PPH, post partum hemorrhage; CS, cesarean section. *p<0.05

Table S2. Association of SES category by paternal education and maternal and perinatal outcomes for pregnant women in Accra, Ghana

|  |  | **Socioeconomic status by paternal education** | | |  | |
| --- | --- | --- | --- | --- | --- | --- |
|  |  | **No education/ primary school** | | **Lower secondary school/ vocational training** | **Senior secondary school/ professional school/ higher tertiary education** | |
|  | **Model** | **OR (95% CI)** | ***P* value** | ***Ref.*** | **OR (95% CI)** | ***P* value** |
| **Maternal outcomes** |  |  |  |  |  |  |
| Hypertensive disorder of pregnancy | Crude | 0.57 (0.13 - 2.52) | 0.46 |  | 1.77 (1.03 - 3.03) | 0.04^a^ |
|  | Adjusted | 0.67 (0.15 - 3.00) | 0.60 |  | 1.71 (0.98 - 2.98) | 0.06 |
| PPH >500 ml | Crude | N.E. |  |  | 0.88 (0.36 - 2.11) | 0.77 |
|  | Adjusted | N.E. |  |  | 0.86 (0.36 - 2.09) | 0.75 |
| Vaginal vs instrumental/CS delivery | Crude | 0.71 (0.26 - 1.88) | 0.49 |  | 0.75 (0.48 - 1.17) | 0.21 |
|  | Adjusted | 0.86 (0.32 0 2.32) | 0.76 |  | 0.73 (0.46 - 1.16) | 0.19 |
|  |  |  |  |  |  |  |
| **Perinatal outcomes** |  |  |  |  |  |  |
| Miscarriage | Crude | 0.76 (0.34 - 1.70) | 0.50 |  | 0.73 (0.50 - 1.07) | 0.11 |
|  | Adjusted | 0.81 (0.36 - 1.82) | 0.60 |  | 0.74 (0.50 - 1.09) | 0.13 |
| Perinatal mortality | Crude | 0.98 (0.12 - 8.31) | 0.99 |  | 0.43 (0.12 - 1.55) | 0.20 |
|  | Adjusted | 0.92 (0.11 - 7.88) | 0.94 |  | 0.45 (0.13 - 1.60) | 0.22 |
| Stillbirth | Crude | 2.98 (0.27 - 33.49) | 0.38 |  | 0.99(0.16 - 5.95) | 0.99 |
|  | Adjusted | 2.77 (0.24 - 32.23) | 0.42 |  | 0.97 (0.16 - 5.85) | 0.97 |
| Low birthweight | Crude | 0.86 (0.25 - 3.00) | 0.81 |  | 1.03 (0.58 - 1.84) | 0.92 |
|  | Adjusted | 0.84 (0.24 - 2.93) | 0.78 |  | 1.04 (0.58 - 1.87) | 0.89 |
| Macrosomia | Crude | 0.57 (0.13 - 2.52) | 0.46 |  | 1.10 (0.62 - 1.95) | 0.76 |
|  | Adjusted | 0.63 (0.14 - 2.80) | 0.54 |  | 1.08 (0.61 - 1.93) | 0.78 |
| Preterm birth | Crude | 0.56 (0.13 - 2.48) | 0.45 |  | 1.19 (0.67 - 2.12) | 0.54 |
|  | Adjusted | 0.59 (0.13 - 2.62) | 0.49 |  | 1.19 (0.67- 2.10) | 0.56 |
| Apgar score < 7  after 5 minutes | Crude | 1.18 (0.25 - 5.57) | 0.83 |  | 0.85 (0.37 - 1.97) | 0.71 |
|  | Adjusted | 1.17 (0.25 - 5.51) | 0.85 |  | 0.86 (0.37 - 1.99) | 0.73 |
|  |  |  |  |  |  |  |
|  |  | **B (CI 95%)** | ***P* value** |  | **B (CI 95%)** | ***P* value** |
| **Birthweight (g)** | Crude | 0.58 (-148.54 - 149.71) | 0.99 |  | 20.09 (-53.70 - 93.87) | 0.59 |
|  | Adjusted | 21.02 (-128.43 -170.48) | 0.78 |  | 19.79 (-53.55 - 93.14) | 0.60 |

OR, odds ratio; PPH, post partum hemorrhage; CS, cesarean section. *p<0.05

Table S3. Association of SES category by employment and maternal and perinatal outcomes for pregnant women in Accra, Ghana

|  |  | **Socioeconomic status by employment** | | |
| --- | --- | --- | --- | --- |
|  |  | **Formal** | | **Informal** |
|  | **Model** | **OR (95% CI)** | ***P* value** | ***Ref.*** |
| **Maternal outcomes** |  |  |  |  |
| Hypertensive disorder of pregnancy | Crude | 1.27 (0.64-2.50) | 0.50 |  |
|  | Adjusted | 1.08 (0.51-2.29) | 0.83 |  |
| PPH >500 ml | Crude | 0.71 (0.16-3.07) | 0.64 |  |
|  | Adjusted | 0.73 (0.17-3.19) | 0.67 |  |
| Vaginal vs instrumental/CS delivery | Crude | 0.94 (0.48-1.84) | 0.86 |  |
|  | Adjusted | 0.90 (0.45-1.82) | 0.78 |  |
|  |  |  |  |  |
| **Perinatal outcomes** |  |  |  |  |
| Miscarriage | Crude | 0.77 (0.42-1.40) | 0.39 |  |
|  | Adjusted | 0.79 (0.44-1.45) | 0.45 |  |
| Perinatal mortality | Crude | 0.70 (0.09-5.49) | 0.73 |  |
|  | Adjusted | 0.70 (0.09-5.53) | 0.73 |  |
| Stillbirth | Crude | 1.40 (0.16-12.11) | 0.76 |  |
|  | Adjusted | 1.53 (0.17-13.43) | 0.70 |  |
| Low birthweight | Crude | 1.22 (0.56-2.67) | 0.62 |  |
|  | Adjusted | 1.23 (0.56-2.70) | 0.60 |  |
| Macrosomia | Crude | 1.17 (0.48-2.84) | 0.74 |  |
|  | Adjusted | 1.23 (0.50-3.01) | 0.65 |  |
| Preterm birth | Crude | 4.56 (0.76-3.20) | 0.23 |  |
|  | Adjusted | 1.61 (0.78-3.32) | 0.20 |  |
| Apgar score < 7  after 5 minutes | Crude | 0.59 (0.14-2.57) | 0.49 |  |
|  | Adjusted | 0.60 (0.14-2.61) | 0.50 |  |
|  |  |  |  |  |
|  |  | **B (CI 95%)** | ***P* value** |  |
| **Birth weight (g)** | Crude | -29.57 (-134.71-75.57) | 0.58 |  |
|  | Adjusted | -16.60 (-121.83-88.63) | 0.76 |  |

OR, odds ratio; PPH, post partum hemorrhage; CS, cesarean section. *p<0.05
